# Molecular Mechanisms Linking Oxidative Stress and Diabetes Mellitus

**DOI:** 10.1155/2020/8609213

**Published:** 2020-03-09

**Authors:** Habib Yaribeygi, Thozhukat Sathyapalan, Stephen L. Atkin, Amirhossein Sahebkar

**Affiliations:** ^1^Research Center of Physiology, Semnan University of Medical Sciences, Semnan, Iran; ^2^Department of Academic Diabetes, Endocrinology and Metabolism, Hull York Medical School, University of Hull, Hull HU3 2JZ, UK; ^3^Weill Cornell Medicine Qatar, Doha, Qatar; ^4^Halal Research Center of IRI, FDA, Tehran, Iran; ^5^Biotechnology Research Center, Pharmaceutical Technology Institute, Mashhad University of Medical Sciences, Mashhad, Iran; ^6^Neurogenic Inflammation Research Center, Mashhad University of Medical Sciences, Mashhad, Iran

## Abstract

Type 2 diabetes mellitus (T2DM) is the most prevalent metabolic disorder characterized by chronic hyperglycemia and an inadequate response to circulatory insulin by peripheral tissues resulting in insulin resistance. Insulin resistance has a complex pathophysiology, and it is contributed to by multiple factors including oxidative stress. Oxidative stress refers to an imbalance between free radical production and the antioxidant system leading to a reduction of peripheral insulin sensitivity and contributing to the development of T2DM via several molecular mechanisms. In this review, we present the molecular mechanisms by which the oxidative milieu contributes to the pathophysiology of insulin resistance and diabetes mellitus.

## 1. Introduction

The prevalence of diabetes mellitus (DM) is growing exponentially worldwide at an epidemic proportion [1]. This chronic disorder has a negative effect on most metabolic pathways and contributes to the pathophysiology of diabetes complications of [2, 3]. Diabetic complications result in considerable morbidity and mortality leading to major healthcare delivery costs [4]. Although there are several studies to elucidate the molecular mechanisms underlying the development of diabetes complications [5–8], their precise pathophysiology is not completely understood [8]. One of the major mechanisms for the development of diabetes complications is through oxidative stress [8]. Oxidative stress develops when the rate of free radical generation exceeds the antioxidant defense systems resulting in the toxic effects of free radicals [9, 10]. Free radical species are important physiological components in biological homeostasis [11, 12], but when their production increases excessively and greater than the body's antioxidant capacity, then oxidative stress results [12]. Oxidative stress is a major upstream event for diabetes complications as well as insulin resistance development [12–14], inducing pathophysiologic molecular mechanisms and initiating a cascade of deleterious pathways leading to insulin resistance and DM [8, 15]. In this review, we discuss the potential roles of oxidative stress in the development of insulin resistance and DM.

## 2. Classification of Diabetes Mellitus

There are different types of DM, but the common subtypes are type 1 DM (T1DM) and type 2 diabetes (T2DM) [16]. T1DM accounts for about 5-10% of all patients with DM which results from beta-cell dysfunction, reduction in insulin release, and lower levels of circulatory insulin [16]. T2DM is the most prevalent type of DM which accounts for about 90-95% of patients with diabetes and is mainly linked to inadequate response to insulin (reduced insulin sensitivity) and insulin resistance in peripheral tissues [16]. Gestational diabetes is another subtype of DM which occurs in pregnant women due to hormonal variations during pregnancy [17]. The other forms of DM are maturity-onset diabetes of the young which is a genetic form of diabetes, latent autoimmune diabetes in adults, and secondary diabetes resulting from other pathologies such as pancreatitis or secondary to the use of medications such as corticosteroids [18, 19].

## 3. Oxidative Stress and Antioxidant Defense System in *β* Cells

Free radicals are active biomolecules which are physiologically generated during metabolic pathways and/or by immune cells [20]. Free radicals have physiological roles in many molecular pathways including those of cellular signaling, synaptic plasticity, memory formation, defense against invader pathogens, cell-cell interactions, cell growth, autophagy, apoptotic processes, and aging [21–24]. When free radical generation increases above the physiological range, it overcomes the antioxidant mechanisms of cells and results in oxidative stress [23, 24]. Most biologic cells have an intrinsic defense mechanism involving various enzymes such as superoxide dismutase (SOD), catalase (CAT), and glutathione (GLT), which protect cells against free radical attack [25].

Free radicals are active derivatives of either the oxygen molecule such as reactive oxygen species (ROS: hydroperoxyl, superoxide, hydrogen peroxide, and hydroxyl radicals) and nitrogen molecules such as the reactive nitrogen species (RNS) peroxynitrite [23]. Some heavy metal derivatives such as iron (ferric) and copper have free radical properties [26]. These hyperactive elements have unpaired electrons in their outer layer of molecules and thereby can bind with other biomolecules and modify them [20, 27]. They can oxidize proteins, lipids, and nucleic acids and produce toxic byproducts leading to tissue dysfunction [27, 28]. Also, they alter the structures of biologic molecules and even break them [28]. DNA breakage is a known effect of oxidative stress, which affects the expression of most genes and cell survival [23]. Free radicals not only have direct deleterious effects, but also can indirectly damage cells by activating a variety of stress-sensitive intracellular signaling pathways such as Nf-*κ*b (nuclear factor kappa b), p38 MAPK (p38 mitogen-activated protein kinases), JNK/SAPK (stress-activated protein kinase/c-Jun NH(2)-terminal kinase), hexosamine pathways, PKC (protein kinase C), AGE/RAGE (advanced glycation end product/receptor for AGE) interactions, and sorbitol synthesis [29]. The various biomarkers for oxidative stress in patients with diabetes include malondialdehyde (MDA), total cholesterol, and reactive hydroperoxides (ROOH) [30]. Oxidative stress has pivotal roles in the pathophysiology of various complications of diabetes through lipid peroxidation, DNA damage, and mitochondrial dysfunction [13, 23, 31, 32]. It is also closely involved in many other pathological conditions as well as age-related disorders such as cardiovascular diseases, chronic obstructive pulmonary disease, chronic kidney disease, neurodegenerative diseases, and cancer [33]. Aging and its related disorders are identified as the progressive loss of tissue function through differing mechanisms including elevated free radical species [33]. Many scientists believe that the oxidative stress theory is the major cause of aging and age-related complications [33]. Hence, maintaining the normal state of redox biology is of importance to prevent oxidative stress-induced complications as well as insulin resistance [34].

## 4. Normal Insulin Signaling Pathways and Insulin Resistance

Insulin resistance is the main underlying pathology in T2DM in which cells are unable to respond to insulin effectively and thereby have a suboptimal uptake of glucose [8, 35]. Normal insulin signal transduction (IST) has complex sequential steps involving different enzymes and mediators, which results in facilitated glucose entry into the adipocytes, muscles, and myocardial cells via GLUT-4 (glucose transporter-4) transporters [8, 34]. The IST is initiated by the binding of insulin to the *α* chain of its specific receptors known as insulin receptors (IRs), which are members of the transmembrane tyrosine kinases composed of *α* and *β* chains and activated by insulin as well as by IGF- (insulin-like growth factor-) 1 and IGF-2 [36]. This binding induces structural changes in the *β* chain by autophosphorylation in tyrosine residues followed by downstream events such as recruitment of different adaptor proteins, i.e., insulin receptor substrates (IRSs), Shc (SHC-transforming) protein, and APS protein (adapter protein with a PH and SH2 domain) [37, 38]. These processes provide an appropriate binding site for the IRS-1 (insulin receptor substrate-1) [38]. Several types of insulin-dependent kinases such as ERK1/2 (extracellular signal-regulated kinase 1/2), atypical PKC (protein kinase C), S6K1 (ribosomal protein S6 kinase beta-1), SIK2 (serine/threonine-protein kinase 2), Akt (protein kinase B), mTOR (mammalian target of rapamycin), and ROCK1 (rho-associated protein kinase 1) and other types of kinases such as AMPK (AMP-activated protein kinase) and GSK-3 (glycogen synthase kinase 3) can phosphorylate IRSs and activate them [38, 39]. Activated IRS-1 binds to PI3K (phosphoinositide 3-kinase) and activates it which, in turn, catalyzes the conversion of PIP_2_ (phosphatidylinositol 4,5-bisphosphate) to PIP_3_ (phosphatidylinositol 3,4,5-trisphosphate) [40]. PIP_3_ is itself a potent activator for Akt, which in turn facilitates glucose entering into the cells by localization of GLUT-4 and by inhibiting glycogen synthase kinase leading to more glycogen synthesis [40, 41]. Any disturbance in these steps can potentially impair normal IST thereby inducing insulin resistance and DM [34].

## 5. Possible Links between Oxidative Stress and Insulin Resistance

As described before, oxidative stress can impair IST and increase the risk of insulin resistance and DM [26, 34]. It must be noted that oxidative stress and DM have complex interactions whereby both intensify each other [42, 43]. In the following sections, we review the possible molecular pathways by which free radicals impair normal glucose homeostasis contributing to the development of DM.

### 5.1. *β*-Cell Dysfunction/Insulin Production and Secretion

A healthy and functional mass of pancreatic beta cells is necessary for normal glucose homeostasis, and DM is accompanied with varying levels of beta-cell dysfunction [44, 45]. A progressive loss of beta-cell mass and function is a major contributor for developing DM [46]. In these conditions, glucose-induced insulin secretion from beta cells becomes deregulated and declines, and therefore, postprandial glucose becomes elevated above the normal [47]. In this process, an initial defect in early or first-phase insulin release occurs, which is then followed by a decreasing maximal capacity of glucose to stimulate postprandial insulin secretion leading to a defective steady-state and basal insulin release followed by complete beta-cell failure [46]. Beta-cell dysfunction results from many pathogenic pathways as well as oxidative stress [46, 48].

Mitochondrial respiratory chains (MRC) and NADPH (nicotinamide adenine dinucleotide phosphate) oxidase or NOX enzyme activity are the major sources of free radicals in the pancreatic beta cells [15, 49–51]. Superoxide anion (O_2_^−^) is the main form of free radical species produced by MRC and NOX enzymes in the beta cells [52]. The phagocytic and immune cells can also produce free radicals that can attack the beta cells [53]. Chronic hyperglycemia induces free radical generation in islets through several molecular pathways such as an increase of cytosolic calcium and protein kinase activation [50, 54]. Since beta cells have a low capacity of the antioxidant defense system, oxidative stress in beta cells is prevalent in DM and plays an important role for the loss of their function in both T1DM and T2DM [45, 48, 55].

**Figure 1:**
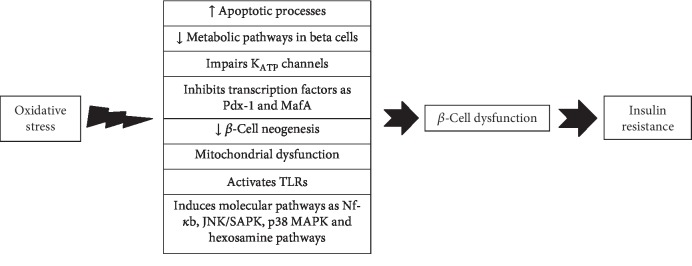
Possible molecular mechanisms between oxidative stress and beta-cell dysfunction leading to diabetes mellitus. Pdx-1: insulin promoter factor 1; MafA: a transcription factor; TLRs: toll-like receptors; Nf-*κ*b: nuclear factor kappa b; p38 MAPK: p38 mitogen-activated protein kinases; JNK/SAPK: stress-activated protein kinase/c-Jun NH(2)-terminal kinase.

Oxidative stress impairs beta-cell function via several molecular mechanisms [48, 55, 56] [57–62]. It markedly reduces insulin production, impairs inclusion of proinsulin vesicles into the plasma membrane, and reduces their exocytosis in response to glucose into the circulation [48, 55, 56]. It can also induce apoptotic processes in the pancreatic cells leading to death and loss of beta cells [48, 55, 56]. A series of proapoptotic agents are highly sensitive to oxidative stress and can activate the apoptotic process in the pancreatic cells [63–65]. Moreover, an overload of free radical species has a negative effect on metabolic pathways in the beta cells and impairs K_ATP_ channels leading to lower insulin secretion [48, 61] (Figure 1). The free radicals impair K_ATP_ channels by binding to their SH residues [57–59], confirmed by studies demonstrating that genetic knockout models of K_ATP_ channels in beta cells resulted in their protection against oxidative stress [61].

Higher concentrations of free radicals inhibit the nuclear transcription factors involved in insulin gene expression as Pdx-1 (insulin promoter factor 1) and MafA (a transcription factor) thereby reducing insulin production at the DNA level [60]. Wang and Wang in 2017 reported that oxidative stress induced molecular pathways such as Nf-*κ*b, JNK/SAPK, p38 MAPK, and hexosamine pathways [62]. These stress-activated signaling pathways have a pivotal role in beta-cell dysfunction [62]. Free radicals can also activate TLRs (toll-like receptors) that in turn impair beta-cell function [66, 67]. Oxidative stress-induced mitochondrial dysfunction in the beta cell is another possible molecular mechanism between oxidative damage and beta-cell dysfunction [48].

Although free radicals have a physiological role in beta-cell proliferation, excess of free radicals will disturb the beta-cell neogenesis [62, 68, 69]. Miceli and coworkers in 2018 found that oxidative stress markedly disturbed beta-cell function in an in vitro experiment [70]. They imposed oxidative stress on rat INS-1E and mouse MIN6 beta-cell lines by exposure to 200 *μ*M hydrogen peroxide (H_2_O_2_) and found that glucose-stimulated insulin secretion was significantly reduced in these cells [70]. Moreover, this event was completely reversed by using carnosine as an antioxidant [70]. Oxidative stress decreases the proliferation and differentiation of beta cells by complex interactions with different factors such as Pdx-1, Nkx6.1, Ngn.3, FOXO, and MafA [62, 71, 72]. These transcriptional mediators are highly sensitive to the redox imbalance, and exposure to higher levels of free radicals negatively modulates the proliferation of the beta cell [71, 72]. Therefore, oxidative stress-induced beta-cell dysfunction is a major pathway where novel therapeutic interventions could be targeted in patients with DM. We suggest that pharmacologic agents protecting islets against oxidative damage can provide a new age of promising therapeutic targets to promote beta-cell function leading to an improvement of glucose homeostasis.

### 5.2. GLUT-4 Expression and/or Localization

As noted above, GLUT-4 regulates glucose entering into the insulin-dependent cells such as adipocytes and myocytes, and therefore, a normal physiological profile of GLUT-4 expression and/or localization is necessary for maintaining insulin sensitivity in these tissues [15, 73]. Any factor which reduces GLUT-4 expression has a marked effect on insulin sensitivity [73, 74] as there is a reduction of glucose entering into the target cells that translates into lower insulin sensitivity in these tissues [75]. Clinical studies show that the GLUT-4 expression and/or localization is lower in patients with insulin resistance and T2DM [74, 76, 77]. This pathophysiologic state is promoted by oxidative stress by the mechanisms noted below [78, 79].

Oxidative stress can reduce GLUT-4 content by negatively effecting its gene expression by impairing the binding of nuclear factor to the insulin responsive element of the GLUT-4 promoter in 3T3-L1 adipocytes [80]. Pessler et al. in 2001 exposed 3T3-L1 adipocytes to micro molar H_2_O_2_ concentrations and developed oxidative stress in these cells and then were detected the Glut-4 expression in these tissues [80]. They found that peroxide hydrogen-induced oxidative stress markedly downregulated GLUT-4 in 3T3-L1 adipocytes and, thereby, reduced glucose entering into the cells [80]. Also, Fazakerley et al. in 2018 conducted a study confirming oxidative stress decreased the GLUT-4 translocation toward the cell membrane [81]. They induced mitochondrial oxidative stress using a mitochondria-targeted paraquat (a selective peroxide generation inducer for mitochondria), in adipocytes and myotubes of mice and observed that oxidative stress markedly suppressed GLUT-4 trafficking and thereby induced insulin resistance in these tissues [81].

Prolonged oxidative stress can suppress the transcriptional factors involved in the GLUT-4 expression such as PPAR-*γ* (peroxisome proliferator-activated receptor gamma), CEB/Ps (CCAAT enhancer-binding proteins), nuclear factor-1, p85, HIF-1*α* (hypoxia-inducible factors alpha), MEF2 (myocyte enhancer factor 2), and Nf-*κ*b [80, 82–84]. It could also suppress micro RNAs involved in the GLUT-4 expression such as miR-21a-5p, miR-222-3p, miR-133b-3p, miR-10b, miR-106b-5p, miR-29c-3p, and miR-133a-3p, although further research is needed to clarify this mechanism [85–88]. Moreover, a wide range of oxidative stress-induced factors and byproducts such as p38 MAPK, JNK/SAPK, PKC (protein kinase C), sorbitol, and hexosamine are all activated by oxidative damage and can suppress GLUT-4 expression [29]. Hence, reduction of GLUT-4 expression/localization is one of the main molecular mechanisms by which oxidative stress induces insulin resistance and contributes to the development of DM [15].

### 5.3. Insulin Signaling Pathways

Any defects in the insulin signaling pathways could potentially contribute to the development of insulin resistance and DM [89, 90]. Modulation of IST has been introduced as a novel therapeutic target for promoting insulin sensitivity [90]. Oxidative stress can impair normal IST at different levels including IRs, IRS-1 and IRS-2; PI3K enzyme; and Akt signaling pathways [91–96]. Balbaa and colleagues in 2017 induced T2DM-induced oxidative stress and then determined IST elements in the brain of diabetic rats [97]. They found that oxidative stress markedly reduced IST element expression as p-IRS, p-AKT, and GSK-3*β* in brain tissues and Nigella sativa oil reversed these events and normalized insulin signaling [97].

Oxidative stress induces IRS-1 and IRS-2 serine phosphorylation, which in turn results in a disturbed IST [91, 92]. Free radicals can induce serine phosphorylation of IRS-1 (at the site of 307) and suppress the normal IST via activation of JNK/SAPK signaling pathways [95]. Moreover, they can inhibit normal IST by p38 MAPK-dependent molecular mechanisms, and inhibition of this molecular pathway restored the normal IST in vitro and in a diabetic animal model [93, 94]. Hyperglycemia-induced oxidative stress activates different stress-sensitive serine/threonine (Ser/Thr) kinases such as IKK-*β* which in turn phosphorylate multiple targets such as the IRs, IRS-1 and IRS-2, leading to unfavorable downstream effects including lower PI3K activation and insulin resistance [29]. It has been shown that salicylates, an inhibitor of IKK-*β*, restored the normal IST in oxidative stress in vitro [98, 99]. Other types of serine/threonine kinases such as Akt (or PKB), GSK-3, AMPK, and mTOR are also very sensitive to oxidative stress and may impair insulin signaling [100–102].

**Figure 2:**
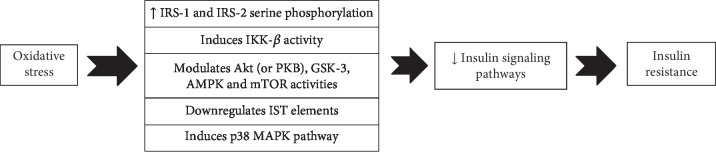
Oxidative stress impairs insulin signaling pathways by several molecular pathways. IST: insulin signal transduction; IRS-1: insulin receptor substrate-1; IKK-*β*: inhibitor of nuclear factor kappa B; GSK-3: glycogen synthase kinase 3; AMPK: AMP-activated protein kinase; mTOR: mammalian target of rapamycin; p38 MAPK: p38 mitogen-activated protein kinases.

Oxidative stress can also result in impairment of IST via downregulation of proteins involved in the normal IST [97]. The main elements of IST such as Akt, IRS, IRS-1, and GSK-3 are under the influence of free radicals that are downregulated by the oxidative stress thereby impairing insulin sensitivity leading to insulin resistance and DM [97]. Hence, impairment of the normal IST is another important link between oxidative stress and insulin resistance [91–96, 100–102] (Figure 2).

### 5.4. Inflammatory Processes

The inflammatory response is one of the main underlying molecular mechanisms involved in the pathophysiology of insulin resistance, DM, and its related complications [53, 103, 104]. There is growing evidence that low-grade chronic inflammation is involved in the pathophysiology of insulin resistance and DM [104–110]. These deleterious processes can also initiate other pathophysiologic mechanisms of DM such as impairment of IST and beta-cell dysfunction [53, 107]. The interaction between inflammatory responses and DM is complex and not fully elucidated [111]. Cytokines can induce Janus kinase pathways (JNKs) which in turn stimulate IRS-1 serine phosphorylation leading to an impairment in IST [112]. These effects were also reported for other inflammatory mediators such as TNF-*α* and Nf-*κ*b which can modulate IRS-1 serine phosphorylation at various sites and thereby impair normal IST [112–114]. Higher levels of monocytes and macrophage activity and circulatory mediators such as CX3CL1 (fractalkine), CRP (C-reactive protein), TNF-*α* (tumor necrosis factor-alpha), IL- (interleukin-) 6, IL-1*β*, IL-18, MCP-1 (monocyte chemoattractant protein-1), resistin, PAI-1 (plasminogen activator inhibitor-1), E-selectin, and IFN-*γ* (interferon-gamma) have been detected in patients with diabetes [104, 106–110]. Therefore, ameliorating these inflammatory processes may be a therapeutic option for the management of diabetes [105, 115, 116].

Many ongoing and/or completed clinical studies have reported the importance of anti-inflammatory agents in glucose homeostasis [117]. For example, Goldfine and coworkers in 2010 evaluated the glucose-lowering effects of salsalate (a prodrug of salicylate) and reported that it was effective in reducing HbA_1c_ and fasting plasma glucose in T2DM patients [117]. Clinical trials that have been undertaken with agents to reduce oxidative stress and their outcomes are shown in Table 1.

Oxidative stress is an upstream event for inflammation as it induces the activation of monocytes and macrophages as well as promotes inflammatory responses involved in insulin resistance and DM [115, 123, 124]. It also upregulates the expression of procytokines and thereby increases the inflammatory mediators at both the mRNA and protein levels [100, 125]. Hence, free radical-induced inflammation is another possible link between oxidative stress and insulin resistance [111].

### 5.5. Systemic Mitochondrial Dysfunction

Mitochondria are double-membrane cellular organelles that have important roles in energy production, calcium storage, fatty acid production, heat production, and cell survival and act as part of cellular signaling pathways [126, 127]. It has been shown that mitochondrial dysfunction is involved in the pathophysiology of insulin resistance and DM; however, the underlying mechanisms remain unclear [128]. Oxidative stress is a key player in most cases of mitochondrial dysfunction [129], impairing mitochondrial function via altering the normal function of the MRC, reducing the respiratory capacity of mitochondria, increasing the proton leak in MRC, altering the potential difference across the inner mitochondrial membrane, and reducing the integrity of mitochondrial membranes [130–132]. These processes may occur locally in the pancreatic islets and/or systemically in adipocytes and muscular tissues [133].

The normal process of glucose uptake via GLUT-4 is closely dependent on the physiological functioning of healthy mitochondria [134] that produce the energy needed for glucose uptake in peripheral tissues [134]. Accordingly, mitochondrial dysfunction markedly reduces the cellular ATP production capacity and impairs the insulin-induced glucose uptake in adipocytes and muscle cells [100]. In these conditions, cells are unable to uptake the circulatory glucose in response to insulin resulting in insulin resistance [100, 133, 135]. In addition, oxidative stress can impair normal mitochondrial function by increasing mitochondrial fatty acid oxidation and DAG (diacylglycerol) production, which in turn activates more serine/threonine kinases leading to impaired IST [100]. Hence, oxidative stress-dependent mitochondrial dysfunction is another molecular mechanism by which free radicals result in insulin resistance [100, 133, 135]. It must be noted that oxidative stress and mitochondrial dysfunction have dual interactions in which both can potentiate each other [136].

## 6. Animal Studies and the Effect of Reducing Oxidative Stress

There is an increasing body of evidence from animal studies confirming oxidative stress-induced insulin resistance and the improvement in IST and glucose homeostasis by using antioxidative agents [93, 94, 132]. Jolivalt and coworkers in 2008 found that high levels of free radical species in brain tissue impaired IST expression and induced insulin resistance in diabetic mice [89]. Agil et al. in 2015 demonstrated that antioxidative properties of melatonin ameliorated insulin resistance by improvement in mitochondrial function in diabetic rats [132]. In addition, Balbaa et al. in 2017 reported that oxidative stress downregulated IST elements in the brain of diabetic animals and demonstrated that it could be reversed by antioxidative agents [97]. Moreover, Zhang and colleagues in 2017 found that myricitrin (a flavonoid) improved insulin sensitivity and glucose homeostasis by attenuating the inflammatory responses and oxidative damage in diabetic mice [123]. Hininger-Favier and coworkers in 2009 evaluated the antioxidative effects of green tea on insulin sensitivity and found that it markedly improved insulin resistance in diabetic rats [137]. Bagul et al. in 2012 reported that antioxidative properties of resveratrol reversed oxidative stress-dependent insulin resistance in diabetic rats [138]. Recently, Sahin et al. in 2019 reported that a phytochemical compound of allyl isothiocyanate significantly increased insulin sensitivity by lowering oxidative stress and inflammatory responses in diabetic rats [139]. Additionally, Abdulmalek and Balbaa in 2019 examined the potential of selenium and found that it promoted insulin sensitivity by ameliorating inflammation and oxidative stress in diabetic rats [140]. These data complement the results of basic studies suggesting oxidative stress induces insulin resistance and DM [97, 123, 132, 137, 139, 140].

Whilst relatively few, there is clinical evidence supporting the animal studies [141]. Udupa et al. conducted a clinical trial on T2DM patients and found that antioxidant agents of omega 3 fatty acids, *α*-tocopherol, and *α*-lipoic acids improved insulin sensitivity in these patients [141]. Manning and coworkers in 2004 reported that high dose vitamin E, a potent antioxidant, transiently improved insulin sensitivity in obese subjects [142]. Ganjifrockwala et al. in 2017 reported that an antioxidant-rich diet decreased oxidative markers accompanied with improved insulin sensitivity in T2DM patients [143]. More recently, van der Schaft and coworkers reported in a large epidemiological study involving 5796 participants that consumption of a diet rich in antioxidants was associated with restoring insulin sensitivity in T2DM patients [144]. These findings suggest that antioxidative agents might effectively improve IST in humans.

## 7. Current Knowledge and Future Perspective

Oxidative stress has a pivotal role in the pathophysiology of insulin resistance and DM, as well as many other complications of diabetes [100, 145]. An increasing number of in vivo studies suggest that higher amounts of free radicals markedly impair normal IST and glucose homeostasis in several ways [100]. Although we have outlined the state-of-the-art knowledge in this field and summarized it into five molecular mechanisms, more cellular pathways may be discovered in the future. More clinical studies using promising antioxidative agents are required to develop new preventive protocols for subjects who are at high risk of insulin resistance, i.e., obese subjects and their place in current therapeutics need to be determined. Antioxidants may play therapeutic roles in reversing insulin resistance in metabolic disorders such as DM, nonalcoholic fatty liver disease, and metabolic syndrome. In addition, a number of herbal-based agents (i.e., crocin, curcumin, cinnamon, and garlic) with confirmed antioxidative properties have potential value in the future [13, 146].

## 8. Conclusion

**Figure 3:**
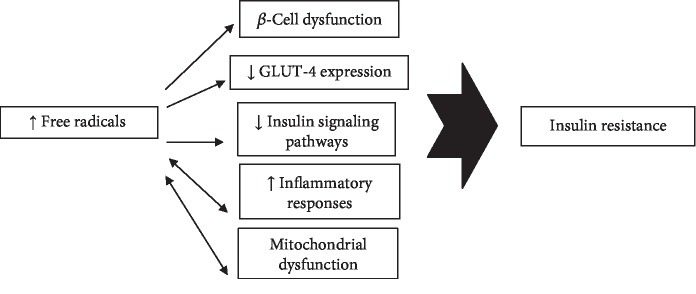
Oxidative stress induces insulin resistance via five major molecular pathways.

Oxidative stress has key roles in the pathophysiology of insulin resistance and DM (Figure 3). It can reduce peripheral insulin sensitivity via at least five major molecular mechanisms through *β*-cell dysfunction, inflammatory responses, GLUT-4 downregulation and/or localization, mitochondrial dysfunction, and impairment of the normal insulin signaling pathways (Table 2). We have described the oxidative milieu imposed by each of these pathways via several molecular mechanisms. Oxidative stress can impair normal IST by negatively modulating the IST elements such as IRS-1 and IRS-2, IKK-*β* activity, Akt, GSK-3, AMPK, and mTOR signaling pathways and p38 MAPK-dependent molecular mechanisms. It can also result in beta-cell dysfunction via induction of apoptotic events, impairing K_ATP_ channels, inhibiting transcription factors involved in *β*-cell neogenesis such as Pdx-1 and MafA, and inducing mitochondrial dysfunction in beta cells leading to lower insulin production/release. Oxidative stress reduces GLUT-4 expression and/or localization by suppressing transcriptional factors such as PPAR-*γ*, CEB/Ps, nuclear factor-1, p85, HIF-1*α*, MEF2, and Nf-*κ*b, as well as various micro RNAs involved in the GLUT-4 expression. Similarly, oxidative stress induces inflammatory responses and beta-cell dysfunction leading to a lower rate of insulin sensitivity in peripheral tissues.

## Figures and Tables

**Table 1:** Clinical trials undertaken with anti-inflammatory agents.

Study population	Study design	Treatment	Dose	Results	Ref.
81 patients with T2DM	Randomized double-blind placebo-controlled trial	Salsalate	3.0, 3.5, or 4.0 g/d for 14 weeks	Reduced the HbA_1c_ and FBG	[117]
20 obese nondiabetic adults	Randomized double-blind placebo-controlled trial	Salsalate	4.0 g/day for 4 weeks	Declined FBG, HbA_1c_, and C-peptide and increased adiponectin	[118]
70 patients with T2DM	Randomized double-blind placebo-controlled trial	Anakinra, a recombinant human IL-1R	13 weeks	Alleviated inflammatory markers and improved glucose control even after treatment withdrawal	[119]
15 patients with T2DM	Open-label trial^a^ and randomized double-blind placebo-controlled trial^b^	Salsalate	3 and 4.5 g/d for 2 weeks	Improved glucose control, circulating free fatty acid, and adiponectin levels	[118]
286 patients with T2DM	Randomized double-blind placebo-controlled trial	Salsalate	3.5 g/d for 48 weeks	Reduced mean HbA_1c_	[120]
257 patients with T2DM	Randomized double-blind placebo-controlled trial	Salsalate	3.5 g/d for 30 months	Reduced inflammatory markers and FBG	[121]
7,000 patients with MI and insulin resistance	Randomized double-blind placebo-controlled trial	Methotrexate	15-20 mg/wk for 3-5 years	Reduced inflammatory markers and HbA_1c_	[122]

Anakinra: a recombinant human IL-1 receptor; FBG: fasting blood glucose; HbA_1c_: glycated hemoglobin; MI: myocardial infarction. ^a^Two studies with open-label design at doses of 3 and 4.5 g/day. ^b^One study with a randomized double-blind placebo-controlled design.

**Table 2:** Main molecular mechanisms by which oxidative stress induces insulin resistance.

Molecular mechanisms	Effects	Ref.
*β*-Cell dysfunction	Induces beta-cell dysfunction through various molecular pathways such as apoptotic events, impairing K_ATP_ channels, inhibiting transcription factors as Pdx-1 and MafA, suppressing *β*-cell neogenesis, and inducing mitochondrial dysfunction in beta cells	[48, 55–62, 71, 72]

GLUT-4 expression and/or localization	Suppresses transcriptional factors involved in GLUT-4 expression as PPAR-*γ*, CEB/Ps, nuclear factor-1, p85, HIF-1*α*, MEF2, and Nf-*κ*b; suppresses micro RNAs involved in GLUT-4 expression	[80, 82–84]

Insulin signaling pathways	Negatively modulates normal IST via IRS-1 and IRS-2, IKK-*β* activity, Akt, GSK-3, AMPK, and mTOR activity and p38 MAPK-dependent molecular pathways	[91–96, 100–102]

Inflammatory events	Increase inflammatory responses which in turn induces insulin resistance in several pathways	[104, 106–109]

Mitochondrial dysfunction	Impairs normal function of mitochondria thereby reduces cellular capacity for glucose uptake by GLUT-4 transporters	[100, 130–133, 135]

PPAR-*γ*: peroxisome proliferator-activated receptor gamma; IST: insulin signaling transduction; CEB/Ps: CCAAT enhancer-binding proteins; HIF-1*α*: hypoxia-inducible factors alpha; MEF2: myocyte enhancer factor 2; IRS-1: insulin receptor substrate-1; Akt: protein kinase B; IKK-*β*: inhibitor of nuclear factor kappa B; GSK-3: glycogen synthase kinase 3; AMPK: AMP-activated protein kinase; mTOR: mammalian target of rapamycin; p38 MAPK: p38 mitogen-activated protein kinases.
